# Treatment of brain abscess rupturing into ventricle: a case report and literature review

**DOI:** 10.3389/fsurg.2026.1782105

**Published:** 2026-06-05

**Authors:** Xiaolin Zhang, Xia Yang, Junan Hu, Wenjing Zhang, Wei Shen

**Affiliations:** 1 Department of Neurosurgery, The Ningbo Beilun District People's Hospital, Ningbo, China; 2 Department of Pharmacy, The Ningbo Beilun District People's Hospital, Ningbo, China; 3 Department of Anesthesiology, The Ningbo Beilun District People's Hospital, Ningbo, China

**Keywords:** brain abscess, external ventricular drainage, metagenomic next-generation sequencing, stereotactic technique, ventriculitis

## Abstract

Brain abscess is a focal intraparenchymal infection. Brain abscess breaking into ventricles is a potentially fatal complication of brain abscess, which can lead to sudden deterioration of neurological function. Its incidence rate is 0.3%–35.0%, and the mortality rate is 84.0%–100.0%. This paper reports a case of a 50-year-old male patient who had previously undergone intracranial hematoma evacuation and skull fixation for traumatic brain injury. He was admitted to the hospital with dizziness for 20 days and bradyphrenia for 4 days. After admission, enhanced computerized tomography(CT) and magnetic resonance imaging(MRI) indicated a left frontal lobe brain abscess. During empirical treatment with ceftriaxone and metronidazole, the abscess ruptured into the ventricle, leading to ventriculitis. Bilateral external ventricular drainage (EVD) combined with ventricular lavage was performed using stereotactic technology. Metagenomic next-generation sequencing (mNGS) of the pus identified the infecting bacteria as Parvimonas micra and Fusobacterium. According to clinical guidelines, the anti-infective regimen was adjusted, short-term low-dose methylprednisolone was used, and combined with hyperbaric oxygen therapy. The patient recovered and was discharged. This paper emphasizes that timely identification of brain abscess rupture leading to ventriculitis, adoption of bilateral external ventricular drainage combined with ventricular lavage, determination of abscess pathogens using mNGS technology, and selection of sensitive antibacterial drugs can improve the cure rate of ventriculitis.

## Introduction

Brain abscess was once a critical neurological condition with a high mortality rate. With the advancement of diagnostic imaging techniques and the establishment of treatment regimens combining surgical drainage with antibiotics, the mortality rate of brain abscess has dropped from 40% to 10% over the past 40 years (1). Rupture of brain abscess into the ventricle leading to ventriculitis is a neurological emergency, often resulting in sudden deterioration of neurological function. As a potentially fatal complication of brain abscess, its incidence ranges from 0.3% to 35.0%, with a mortality rate of 84.0% to 100.0% (2). Recently, our department successfully treated a patient with brain abscess complicated by ventriculitis due to rupture. Through early identification, stereotactic puncture with ventricular drainage combined with ventricular irrigation, administration of sensitive antibacterial agents, nutritional support, hyperbaric oxygen therapy, and other comprehensive treatments, the patient achieved complete recovery. By reviewing the treatment process and relevant literature, this report aims to improve clinicians’ diagnostic and therapeutic capabilities for such patients.

## Case presentation

**Figure 1:**
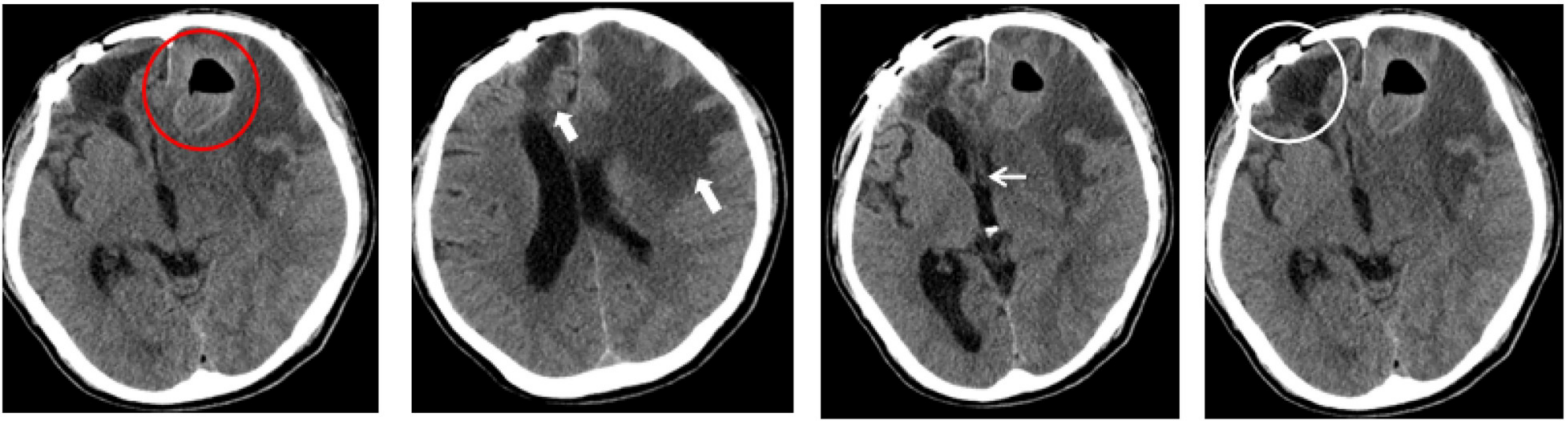
Pre-hospital CT: A unilocular cystic lesion is seen in the left frontal lobe, with gas inside the cyst and mixed-density cystic fluid (red circle); edema of the frontal lobe (thick arrow), marked midline shift (thin arrow); postoperative changes of the right frontal bone (white circle).

The patient was a 50-year-old male who was admitted to the Department of Neurosurgery, Beilun People's Hospital on August 21, 2024, with the chief complaints of dizziness for 20 days and bradyphrenia for 4 days. Pre-admission CT scan showed heterogeneous density shadow in the left frontal lobe, extensive edema in bilateral frontal lobes, slight rightward deviation of midline structures, and postoperative changes of the right frontal bone (Figure 1).

The patient suffered traumatic brain injury due to a traffic accident in 2012 and underwent craniotomy for intracranial hematoma evacuation and skull fixation. He had a good postoperative recovery. He denied a history of diabetes mellitus, immunosuppressant use, and alcohol abuse.Neurological examination revealed the patient had bradyphrenia and cognitive impairment. According to the Lovett muscle strength scale, the muscle strength of the right limb was grade IV and that of the left limb was grade V. No meningeal irritation signs were observed. After admission:an emergency lumbar puncture was performed for cerebrospinal fluid (CSF) examination. The CSF was clear, with a white blood cell (WBC) count of 20 cells/μL, neutrophil ratio of 36%, glucose level of 58.68 mg/dL, and protein level of 440.7 mg/L. Peripheral blood test showed WBC count of 12.2 × 10^6^ cells/μL and neutrophil count of 8.8 × 10^6^ cells/μL. Serum sodium level was 125.7 mmol/L.

Admission diagnoses included intracranial space-occupying lesion suspected as left frontal lobe brain abscess, hyponatremia, hemiplegia, and cognitive impairment.

**Figure 2:**
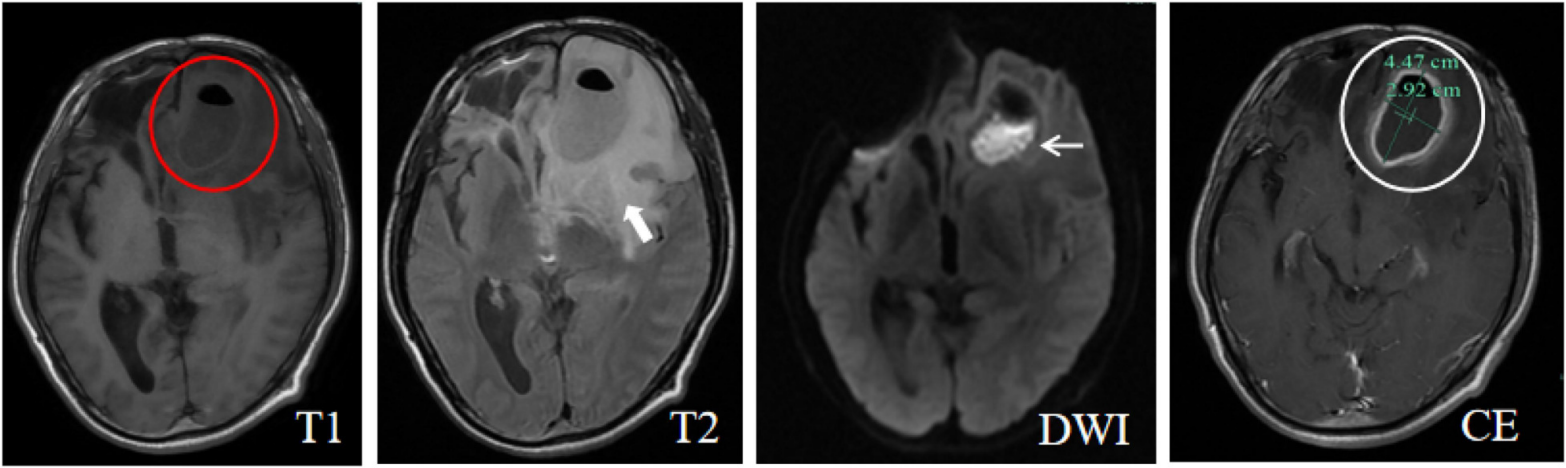
Contrast-enhanced cranial MRI: A cystic space-occupying lesion is visualized on the T1-weighted sequence, with the density of the cyst fluid higher than that of cerebrospinal fluid and gas present inside the cyst (red circle); extensive cerebral edema is observed around the cystic space-occupying lesion on the T2-weighted sequence (thick arrow); the cyst fluid shows hyperintensity changes on the DWI sequence (thin arrow); and ring enhancement of the cyst wall of the space-occupying lesion is seen on the contrast-enhanced sequence, with a size of 4.47 cm × 2.92 cm(white circle).

**Figure 3:**
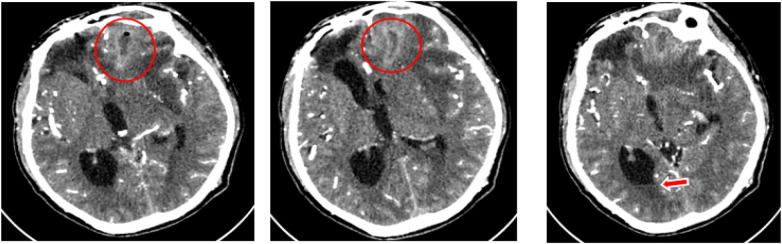
Contrast-enhanced cranial CT: rupture of the brain abscess with significant reduction in volume (red circle); empyema is visualized in the posterior horn of the ventricle (red arrow).

**Figure 4:**
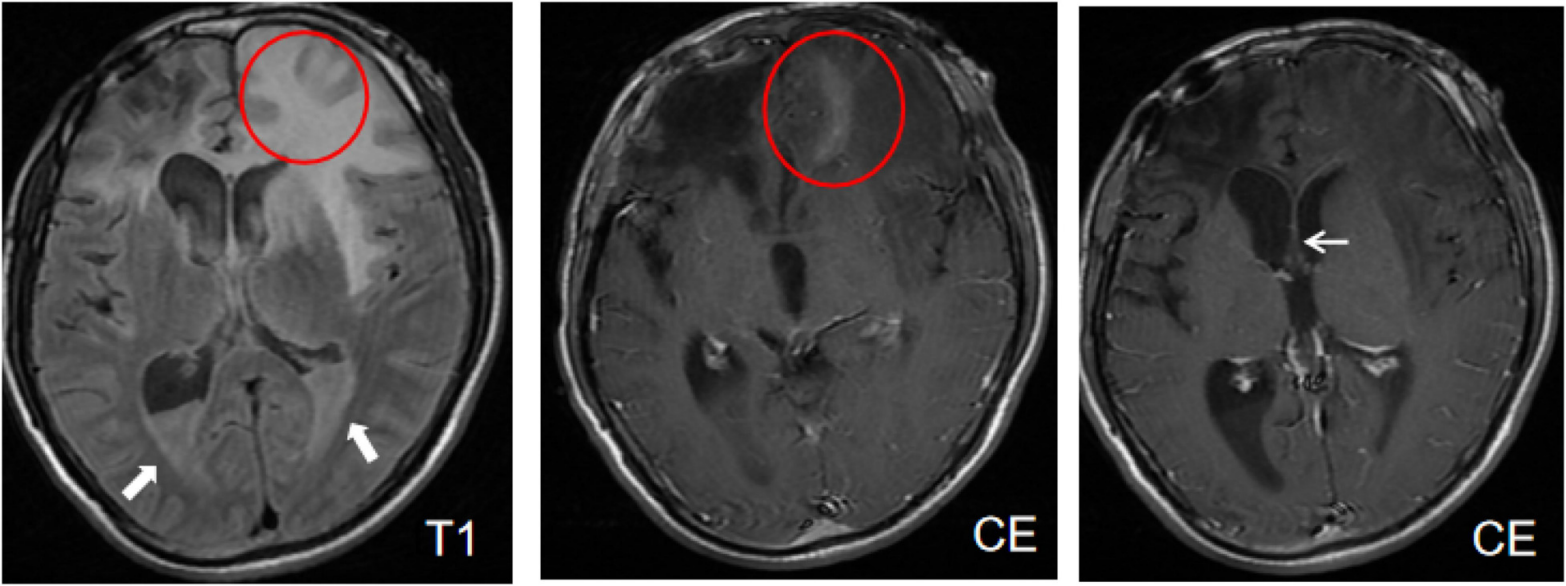
Cranial MRI: rupture of the brain abscess is visualized on T1-weighted and contrast-enhanced (CE) sequences, with only the abscess wall remaining (red circle); empyema is present in the posterior horns of the bilateral ventricles (thick arrow); the midline shift is significantly reduced (thin arrow).

**Figure 5:**
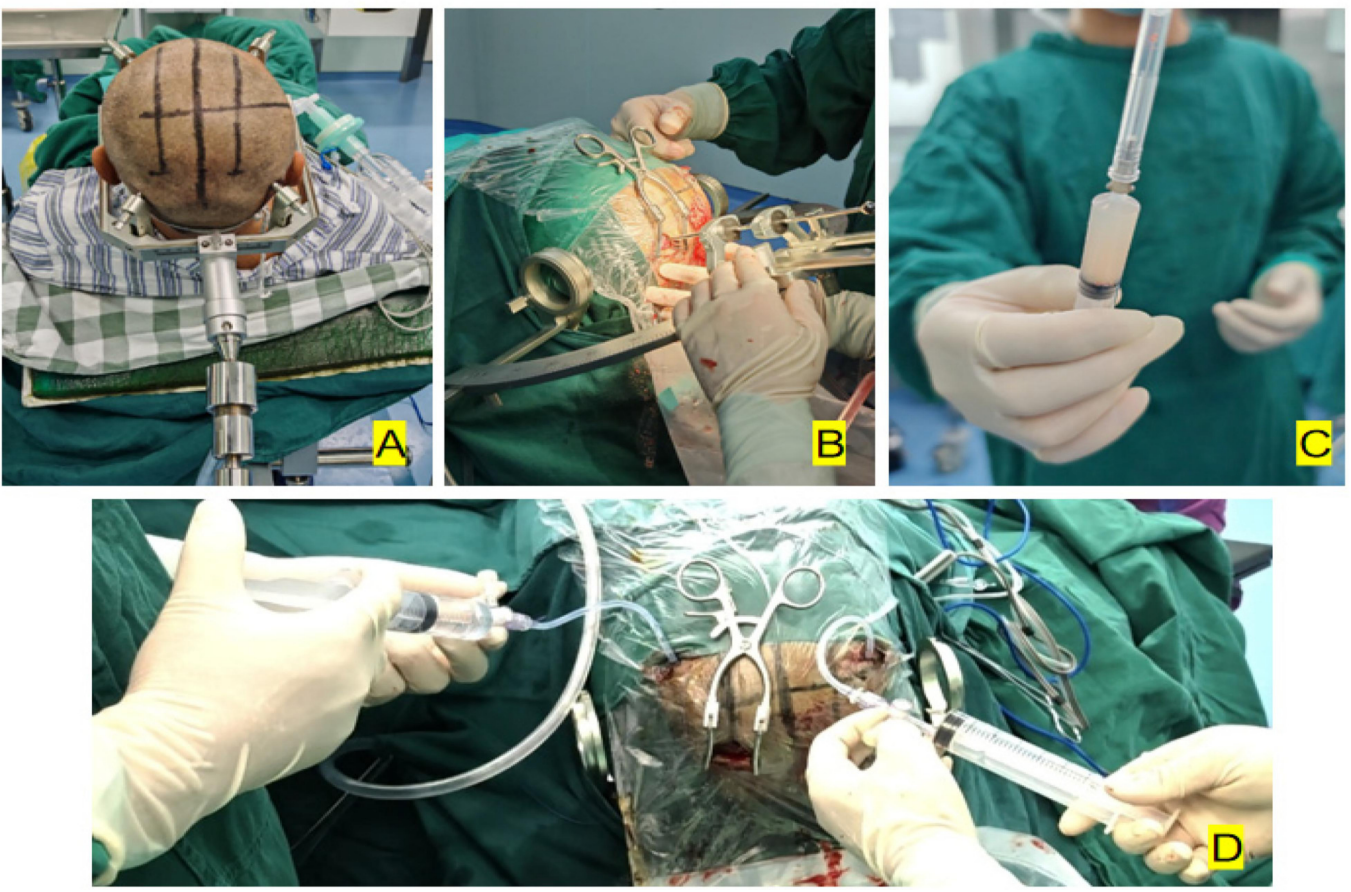
Surgery: head position with stereotactic frame fixation **(A)**; stereotactic-guided ventricular empyema aspiration **(B)**; empyema aspirated from the ventricle **(C)**; ventricular irrigation **(D).**

**Figure 6:**
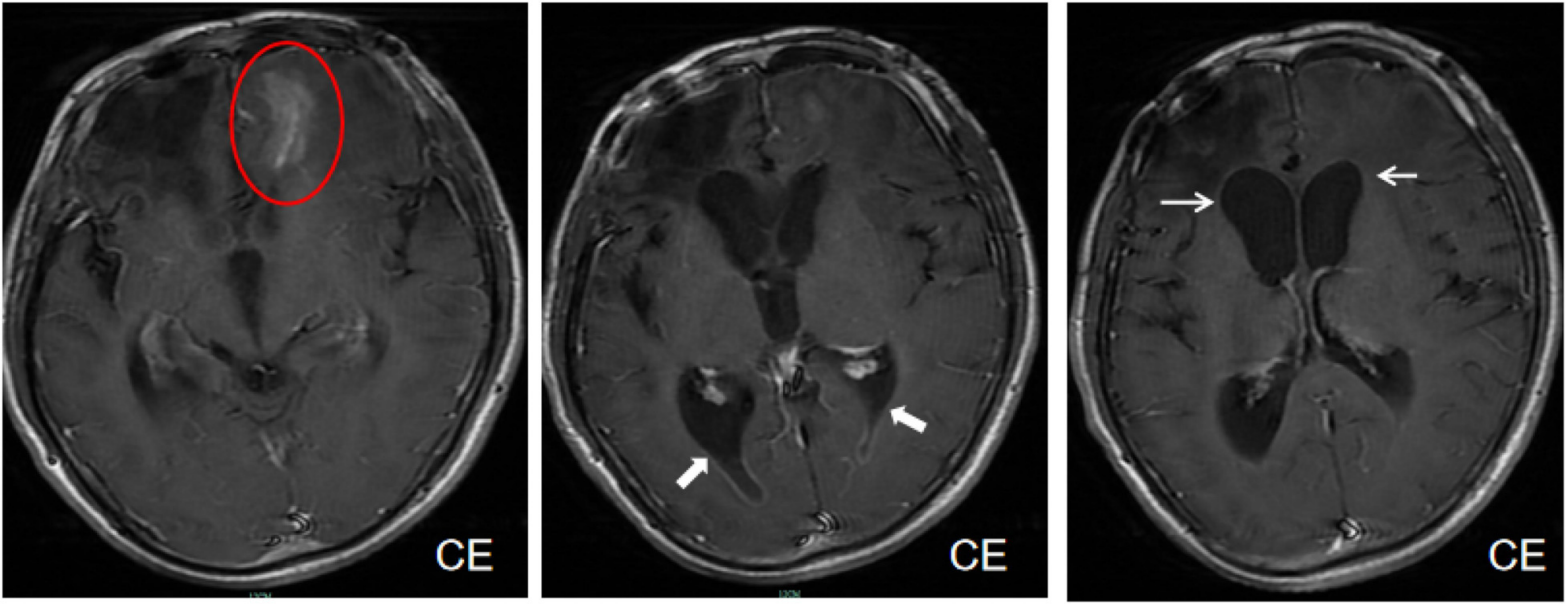
Contrast-enhanced cranial MRI: residual abscess wall is noted (red circle); no empyema (thick arrow)or hydrocephalus (thin arrow) is identified in the cerebral ventricles.

Initial treatment consisted of intravenous ceftriaxone 2.0 g once daily combined with intravenous metronidazole 0.5 g every 8 h for anti-infective therapy, intravenous 20% mannitol 100 mL every 8 h for dehydration and intracranial pressure reduction, intravenous methylprednisolone 80 mg once daily to alleviate cerebral edema, intravenous sodium valproate 0.8 g twice daily for seizure prophylaxis, and enteral nutrition of 1500 mL daily via nasogastric tube for nutritional support. A contrast-enhanced cranial MRI was completed to further confirm the nature of the space-occupying lesion as brain abscess (Figure 2).The patient was scheduled to undergo stereotactic brain abscess puncture and drainage. During preoperative preparation, 32 h after admission, the patient developed sudden hyperthermia with a body temperature of 39.4℃, without vomiting, convulsion, or disturbance of consciousness. An emergency contrast-enhanced CT scan suggested brain abscess rupture into the ventricle leading to ventriculitis (Figure 3). A repeated lumbar puncture was performed, and the CSF appeared cloudy, with a WBC count of 2690 cells/μL, neutrophil ratio of 80%, glucose level of 34.56 mg/dL, and protein level of 1025.5 mg/L. CSF samples were sent for culture and mNGS. The CSF examination confirmed the diagnosis of ventriculitis.One day after lumbar puncture,metagenomic testing identified the pathogens as Parvimonas micra and Fusobacterium. On day 4 after admission,the anti-infective regimen was adjusted to ceftriaxone 4.0 g daily by intravenous infusion plus metronidazole 0.5 g every 8 h by intravenous infusion in accordance with clinical guidelines (3, 4). A re-examination of contrast-enhanced cranial MRI revealed empyema in the posterior horns of bilateral lateral ventricles (Figure 4). Under endotracheal intubation and general anesthesia, the patient underwent stereotactic-guided puncture and drainage of the posterior horns of the bilateral lateral ventricles. Intraoperatively aspirated pus was submitted for microbial culture and mNGS, and the ventricular system was extensively irrigated with warm normal saline (Figure 5). Postoperatively, the patient received intravenous ceftriaxone 4.0 g daily plus metronidazole 0.5 g every 8 h for anti-infection over 15 days. Additional therapy included 20% mannitol 100 mL every 8 h for intracranial pressure control, methylprednisolone 80 mg daily for cerebral edema, and sodium valproate 0.8 g twice daily for seizure prophylaxis. Enteral nutrition 1500 mL daily was delivered via nasogastric tube.During EVD, lumbar puncture was performed 2 h after drain clamping to slowly release 30∼40 mL CSF, with the drain reopened 2 h later; this cycle was repeated 3 times to restore CSF circulation. The patient's body temperature, CSF white blood cell count, and glucose level gradually normalized. CT confirmed patency of the CSF pathway, and lumbar puncture demonstrated normal intracranial pressure on three measurements.One week postoperatively, the left ventricular drain was removed. The right drain was gradually elevated to reduce CSF output, and was subsequently removed after 3 days of afebrile status, no CSF leakage, and no signs of intracranial hypertension. On postoperative day 15, contrast-enhanced MRI showed resolution of ventricular empyema and no hydrocephalus (Figure 6), and the patient was discharged.

**Figure 7:**
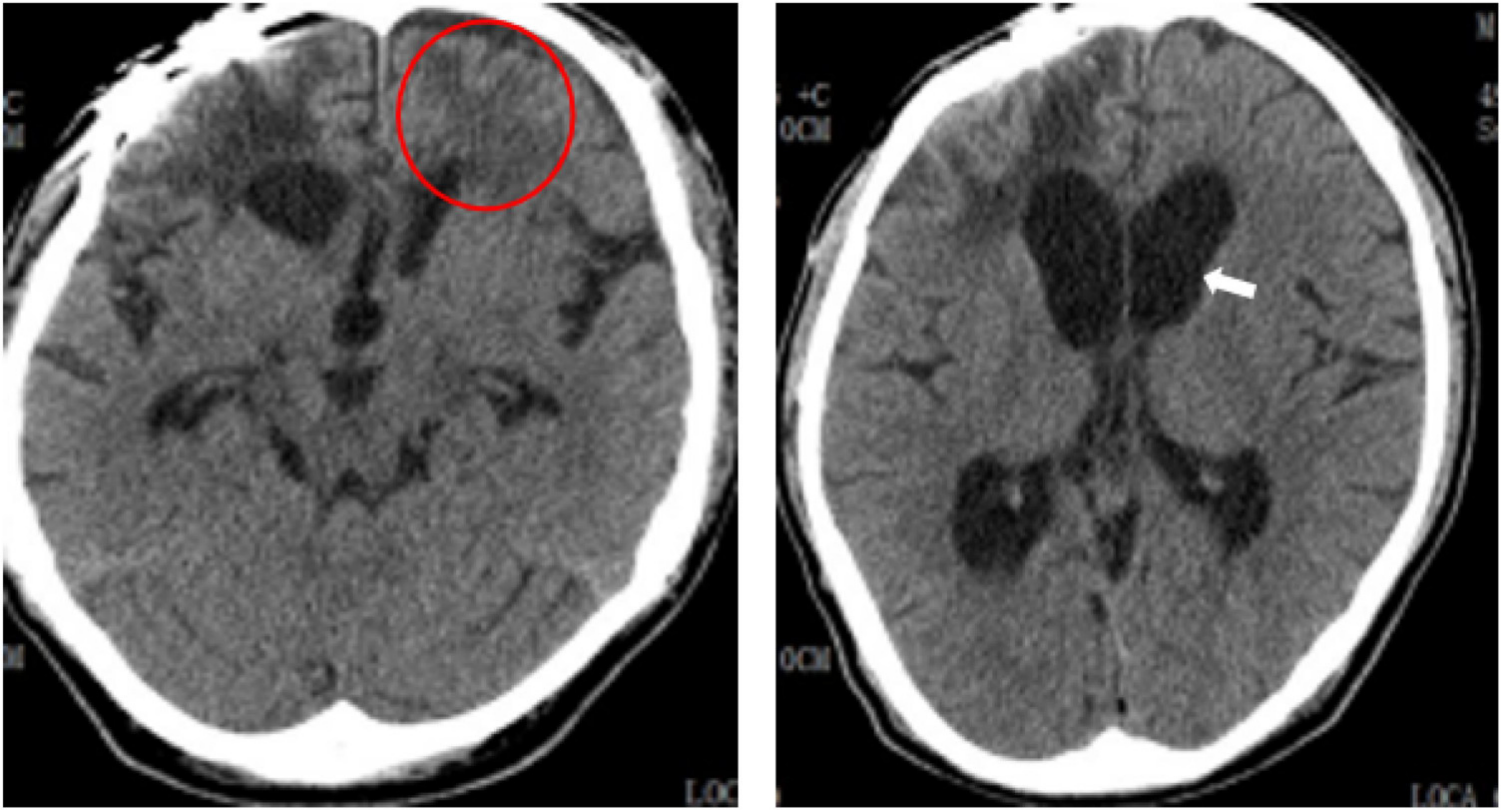
Cranial CT: the brain abscess has resolved (red circle); no progressive hydrocephalus changes are noted (thick arrow).

After discharge, the patient completed 36 sessions of hyperbaric oxygen therapy at 2 atmospheres absolute for 95 min per session and continued intravenous ceftriaxone and metronidazole for 6 weeks. Inflammatory markers remained normal, and follow-up CT confirmed abscess resolution without hydrocephalus (Figure 7). The patient was alert with fluent speech and grade 5 muscle strength in all limbs, achieving full recovery.

## Discussion

Brain abscesses can be caused by bacteria, fungi, or parasites nested as encapsulated areas of pus within the cerebral parenchym. The clinical symptoms of patients with brain abscess are caused by increased intracranial pressure and mass effect, and sometimes by focal brain injury. Headache, fever, and focal neurological deficits constitute the classic triad of brain abscess; however, the symptoms are usually non-specific in the early stage of the disease. According to reports, only 7% of patients present with the classic triad in the early stage, which makes misdiagnosis easy (5). Contrast-enhanced cranial MRI is the most sensitive examination for detecting and localizing brain abscesses, as it can reveal the typical ring-enhancing lesions and hypodense abscess cavities of brain abscesses (6). In addition, MRI diffusion-weighted imaging (DWI) and apparent diffusion coefficient (ADC) are of great significance in differentiating encapsulated brain abscesses from necrotic and cystic intracranial tumors: brain abscesses show hyperintensity on DWI and hypointensity on ADC, whereas the necrotic and cystic areas of intracranial tumors show hypointensity on DWI and hyperintensity on ADC (7).

The clinical treatment of brain abscesses is mainly divided into two categories: conservative treatment and surgical treatment. Antibacterial agents are the cornerstone of brain abscess treatment. In the early stage of abscess formation or when the abscess is small, targeted antibacterial therapy can be administered based on drug sensitivity test results. For patients with brain abscess, CSF culture is often negative. The positive rate can be improved by collecting CSF for culture 2–3 consecutive times or performing culture on aspirated pus. In addition, if CSF or pus culture is negative, mNGS can be conducted to detect potential pathogenic bacteria (8). Empirical medication is usually adopted before the pathogenic bacteria of brain abscess are identified.For community-acquired brain abscesses, the most common pathogenic bacteria are oral flora, such as Streptococcus anginosus group, Fusobacterium and Aggregatibacter, which are usually associated with dental and chronic ear infections. European guidelines recommend third-generation cephalosporins combined with metronidazole as empirical antibacterial therapy for such brain abscesses (9). In the case reported here, before the definite pathogenic bacteria were identified, the patient was treated with ceftriaxone 2.0 g daily by intravenous infusion plus metronidazole 0.5 g every 8 h by intravenous infusion. Both CSF and pus mNGS indicated Parvimonas micra or Fusobacterium. According to the drug sensitivity results, the treatment was continued with ceftriaxone 4.0 g daily by intravenous infusion plus metronidazole 0.5 g every 8 h by intravenous infusion, which showed a good antibacterial effect during the treatment course.Surgical treatment is indicated for brain abscesses under the following circumstances (10): abscess diameter > 2.5 cm with mass effect or even brain herniation,risk of rupture into the ventricle, failure of medical treatment, Fungal infection, neurological deficit, multiloculated abscess. The surgical methods can be abscess puncture and drainage or craniotomy for abscess resection. For patients with a skull window, abscess puncture can be performed under ultrasound guidance.For brain abscesses completely resected by surgery, the course of antibacterial treatment should be 4–6 weeks; for those treated with puncture and drainage, the course should be 6–8 weeks; and for those receiving conservative treatment alone, the course should be longer. Researchers found that hyperbaric oxygen therapy can significantly shorten the duration of antibiotic use, improve treatment efficacy and ameliorate neurological function in patients with brain abscess who are treated with stereotactic puncture and aspiration combined with antibiotics (11).

Rupture of brain abscess into the ventricle resulting in ventriculitis is a potentially fatal complication of brain abscess. It is more common than rupture of brain abscess into the subarachnoid space, which may be attributed to the fact that the capsule on the cortical side is more intact than that on the ventricular side during abscess formation due to differences in blood supply (12). Rupture of brain abscess into the ventricle can lead to severe ventriculitis, which may progress to extensive panmeningoencephalitis if treatment is delayed. Therefore, early identification of brain abscesses and those prone to rupture into ventriculitis plays a crucial role in improving patient prognosis. Known risk factors for rupture of brain abscess into the ventricle include multiloculated morphology and a short distance between the ventricular wall and the abscess, whereas abscess size is not a risk factor. Studies have shown that the risk of brain abscess rupturing into the ventricle during hospitalization in patients with multiloculated brain abscesses is 4.2 times that in patients with uniloculated brain abscesses. For every 1 mm reduction in the distance between the ventricle and the brain abscess, the risk of rupture increases by 10% (13). Brain abscesses located in the parieto-occipital region have a higher risk of rupture. In addition, brain abscesses caused by hematogenous spread are usually located at the gray-white matter junction with poorly formed capsules, thus being more likely to rupture into the ventricle (14). The high-risk factors for ventriculitis in this case were as follows:close proximity between the abscess and the ventricular wall,presence of gas-producing bacteria in the abscess, increased pressure gradient between the abscess and the ventricle after lumbar puncture. For patients with such brain abscesses that are prone to rupture and cause ventriculitis, early stereotactic puncture and aspiration under effective anti-infective treatment after admission can significantly reduce the occurrence of this severe complication ventriculitis.

Rupture of the abscess into the ventricle can cause patients to present with sudden hyperthermia, severe headache, frequent vomiting, and epilepsy; in severe cases, patients may lapse into a comatose state. The optimal treatment strategy for patients with brain abscess rupturing into the ventricle has not yet been determined. A systematic review of surviving patients with brain abscess rupturing into the ventricle found that, except for a very small number of patients who received only medical treatment, the vast majority underwent surgical intervention combined with systemic intravenous and/or intraventricular antibiotic administration. The most common surgical procedure was EVD, continuous drainage helps control increased intracranial pressure caused by hydrocephalus, reduce intraventricular pus and debris, and can serve as an administration route for intraventricular antibiotics. The second most common procedure was burr hole drilling and abscess aspiration, while a small number of patients underwent treatment strategies including craniotomy, abscess cavity debridement, and ventricular irrigation (2). Precise placement of an external ventricular drainage tube under stereotactic guidance not only facilitated maximum aspiration of empyema in the posterior horn of the ventricle during surgery and continuous irrigation with normal saline intraoperatively but also ensured unobstructed continuous drainage postoperatively, which was conducive to the external drainage of purulent CSF. During external CSF drainage, intermittent lumbar punctures were performed to release purulent CSF, which promoted CSF clearance, early restoration of unobstructed CSF circulation, reduced the incidence of hydrocephalus, and ensured the patient safely passed through the acute phase of ventriculitis.

Whether glucocorticoids should be included in the treatment of brain abscesses has long been controversial. Some studies (15, 16) have suggested that glucocorticoids can induce various adverse effects, such as reduced antibiotic permeability, impaired leukocyte migration, weakened host defense mechanisms, slowed pathogen clearance, disrupted capsule formation, and diminished ring enhancement on imaging. However, other studies have indicated that glucocorticoids can inhibit immune-mediated inflammation in brain tissue induced by bacterial infection, thereby reducing abscess volume and improving patient prognosis. Literature reports have shown that patients with increased intracranial pressure can benefit from short-term use of high-dose corticosteroids, for example:intravenous administration of 10 mg dexamethasone as a loading dose, followed by 4 mg intravenously every 6 h for 3 to 4 days (15). In addition, a good nutritional status is also conducive to the treatment of brain abscesses and ventriculitis.

## Conclusions

In summary, rupture of brain abscess into the ventricle resulting in ventriculitis is a potentially fatal complication of brain abscess and constitutes a neurological emergency (17). Early identification of brain abscess, timely recognition of ventriculitis, improved pathogen detection rate via mNGS technology, intravenous administration of sensitive antibacterial agents, short-term low-dose methylprednisolone to reduce inflammatory response and cerebral edema, implementation of bilateral EVD combined with ventricular irrigation, and adequate nutritional support,can often improve the treatment outcomes for brain abscess ruptured into ventriculitis.

## Ethics statement

Written informed consent was obtained from the individual for the publication of any potentially identifiable images or data included in this article.
